# The Editor's Letter-Box

**Published:** 1912-03-23

**Authors:** W. Milburn


*To the Editor of* The Hospital.

Sir,—With reference to the interesting correspondence now appearing in your columns *re* recent hospital competition awards, I notice that one of the principal objections raised by Mr. G. L. D. Hall to the Bradford award is the statement that the author of the winning design had treated the site as if it were level, whereas there is a considerable fall across it. I do not think, however, that Mr. Hall's informant can have seen the actual competition designs or that the statement is a correct one on which to criticise the award. I was also a competitor, and after the award had the pleasure of inspecting the whole of the designs when on exhibition at Bradford. The winning design was most carefully worked out in every detail, and a block section above the block plan clearly showed the treatment of the levels of the site, and the necessary excavation and terracing, while the sixteenth-scale sections further elucidated the scheme.—Yours faithfully,

W. Milburn, Jun.

